# Declining regional disparities in mortality in the context of persisting large inequalities in economic conditions: the case of Germany

**DOI:** 10.1093/ije/dyz265

**Published:** 2020-01-16

**Authors:** Alyson A van Raalte, Sebastian Klüsener, Anna Oksuzyan, Pavel Grigoriev

**Affiliations:** 1 Max Planck Institute for Demographic Research, Rostock, Germany; 2 Federal Institute for Population Research, Wiesbaden, Germany; 3 Vytautas Magnus University, Kaunas, Lithuania

**Keywords:** mortality, life expectancy, inequalities, variation, area analysis, economic status

## Abstract

**Background:**

Subnational regional mortality inequalities are large and appear to be mostly increasing within industrialized countries, although comparative studies across high-income countries are scarce. Germany is an important country to examine because it continues to experience considerable economic disparities between its federal states, in part resulting from its former division.

**Methods:**

We analyse state-level mortality in Germany utilizing data from a newly constructed regional database based on the methodology of the Human Mortality Database. We compare time trends (1991–2015) in the German state-level standard deviation in life expectancy to that of other large, wealthy countries and examine the association between mortality and economic inequalities at the regional level. Finally, using contour-decomposition methods, we investigate the degree to which age patterns of mortality are converging across German federal states.

**Results:**

Regional inequalities in life expectancy in Germany are comparatively low internationally, particularly among women, despite high state-level inequalities in economic conditions. These low regional mortality inequalities emerged 5–10 years after reunification. Mortality is converging over most ages between the longest- and shortest-living German state populations and across the former East–West political border, with the exception of an emerging East–West divergence in mortality among working-aged men.

**Conclusions:**

The German example shows that large regional economic inequalities are not necessarily paralleled with large regional mortality disparities. Future research should investigate the factors that fostered the emergence of this unusual pattern in Germany.

**Key Messages**

- Among large Organisation for Economic Cooperation and Development (OECD) countries, Germany went from having the highest observed regional inequalities in mortality at the time of reunification to now having the lowest regional inequalities in mortality for women and moderate inequalities for men.
- The low German regional inequalities in life expectancy coexist with large and persisting regional inequalities in economic conditions.
- Although convergence is observed for overall mortality, an emerging East–West divergence in mortality at younger adult ages among men could presage future divergence in life expectancy.

## Introduction

*Now what belongs together, will grow together* (Willy Brandt).

Following the collapse of the Berlin Wall, Brandt’s famous quote aspired to a convergence of living conditions in what 1 year later would become a unified Germany. Whereas substantial progress has been made in East vs West economic convergence since the 1990 German reunification, considerable disparities continue to exist between the formally divided halves.^1–3^ The states of the former East Germany (hereafter eastern Germany) are still poorer, and since the fall of the Berlin Wall more than 30 years ago, have experienced high levels of structural unemployment.^4^ Life-expectancy convergence, on the other hand, has been touted as a major success story.^5–7^ Females in eastern Germany now have a similar (even slightly higher) life expectancy at birth compared with their counterparts from western Germany, whereas, among men, the gap has substantially narrowed but has not been eliminated.^8^^,^^9^

While many studies have looked at East–West mortality differences in Germany, the levels and trends at lower spatial aggregations such as states and districts remain less well understood. In part this is because long-term data at these levels are often difficult to obtain due to frequent reforms of the administrative divisions.^10^ In addition, existing analyses^11–14^ are usually based on the uncorrected official data, which, in the 1990s and 2000s, suffered from notable numerator/denominator biases.^15^

Outside of Germany, regional inequalities in mortality appear to mostly be on the increase since at least the turn of the century. In the USA, increasing inequality in life expectancy has been observed at both the county^16^^,^^17^ and state levels.^18^ In the UK, inequalities across districts grouped into quintiles of deprivation increased in England and Wales^19^ and in Scotland.^20^^,^^21^ In France, mortality inequalities across the *départements* declined markedly throughout much of the twentieth century, but have since increased among men and remained stable among women.^22^^,^^23^ In Russia, the population-weighted standard deviation in life expectancy experienced large increases and sharp fluctuations during the mortality crises of the 1980s and 1990s, before stabilizing in the mid-2000s.^24^ Some smaller countries have reported stagnation or small declines in regional mortality inequalities, including Canada (1980–2007),^18^ the Netherlands (1988‒2009),^25^ Austria (1969–2004)^26^ and the Czech Republic (1991–2015).^27^

These single-country studies are difficult to compare because of differences in the outcome measure (life expectancy, age-standardized death rates), age ranges examined, time periods, the size of the geographic unit and the measure of inequality or dispersion. Studies comparing regional inequalities across countries are scarce. To our knowledge, the only cross-country study of regional inequalities in mortality analysed trends in the standard deviation in remaining life expectancy at age 50 years across comparably large regions in five countries, from 1921 to 2007.^28^ Germany was one of the countries investigated (from 1990 to 2007) and demonstrated moderate but declining regional inequality. However, the focus on older adult mortality excluded key ages for which we might expect regional mortality differences to be most salient.

Mortality inequalities are important to examine because they are generally avoidable, and often thought to result from an unequal distribution of resources or opportunities.^29^ At the country level, national income and life expectancy have long been strongly positively correlated in the cross-section.^30^ Existing evidence suggests that this also holds true at more fine-gridded regional levels.^11^^,^^31^ In a multilevel framework, the distribution of economic conditions across US states was found to be an important determinant of variation in women’s mortality across US states.^32^ Germany experiences comparatively large regional differences in living conditions, income per capita and unemployment rates.^33^^,^^34^ As such, we expected to find substantial inequalities in mortality between German *Länder* (hereafter states), particularly compared with other European countries.

Here, we present analyses of state-level mortality from a newly constructed and harmonized regional database. In what follows, we assessed trends in subnational regional-level inequalities in mortality beginning from the decade prior to reunification. We compared the magnitude of subnational regional-level inequalities in mortality to those existing in other large wealthy countries, explored the relationship between economic conditions and subnational regional-level mortality in these countries, and examined the degree to which age patterns of mortality, in addition to life expectancy, are converging across German state borders.

## Data and methods

### The German Regional Human Mortality Database (HMD), 1982–2015

Our time series covers an unusually long intercensal period between the 1981 census of East Germany, the 1987 census of West Germany and the first census after reunification in 2011. The 2011 census revealed that the pre-existing official population counts were overestimated by 1.5 million individuals (out of a total population of 80.3 million). As the German statistical offices did not produce adjusted intercensal population estimates by age, we had to develop and apply our own methodology for data harmonization.^15^ Period life tables by sex, year and single year of age (0‒110+) were calculated using the HMD methodology^35^^,^^36^ for the period 1982–2015. Because of differences in the definition of live births in East Germany prior to reunification,^37^ we make all comparisons from age 5 years onwards. Further details about the German Regional HMD and its sources are provided in the Online Supplementary Appendix A, available as Supplementary data at *IJE* online.1

### Additional data and methods

As a measure of dispersion in outcome measures across regions, we use the standard deviation, which measures absolute inequality and is easy to interpret. Time trends in the German state-level standard deviation (*SD*) in remaining life expectancy at age 5 years (*e*_5_) and in per-capita GDP were compared with equivalent state-level inequalities from all Organisation for Economic Cooperation and Development (OECD) countries with 30 million inhabitants or more, which had mortality data freely available and of a sufficient quality at the subregional level. For European countries, these time trends were compared at the Nomenclature of Territorial Units for Statistics (NUTS) 1 level, which generally corresponds to population sizes ranging from 3 to 7 million inhabitants. For non-European countries, we used nationally defined subnational regional territorial units.

Life expectancy can increase smoothly despite stalls or increases in mortality over working ages, to which life expectancy is not particularly sensitive.^38^ Emerging disparities at these ages could portend future life-expectancy divergence if such disparities were cohort-patterned, i.e. following individuals of specific birth cohorts into older ages. To verify the extent to which life-expectancy convergence was accompanied by convergence in the full age pattern of mortality, we employed contour decompositions.^39^ This method splits the age-specific mortality contributions to a current *e*_5_ gap between two populations into: (i) contributions from age-specific mortality differences at an initial time period and (ii) contributions from different age-specific mortality trends from the initial to final time periods. In other words, it is a method to directly compare mortality convergence across age, while controlling for initial age-specific mortality differences.

To narrow the variation in age-specific mortality patterns across our 17 states (former East and West Berlin are treated separately) into a few meaningful groupings for contour decomposition, we analysed: the three states with the lowest *e*_5_ in eastern Germany (*East Low*), the three states with the lowest *e*_5_ in western Germany (*West Low*) and the three states with the highest *e*_5_ in eastern Germany (*East High*) (see Online Supplementary Appendix A, available as Supplementary data at *IJE* online for details). As a reference for all of our comparisons, we chose the German low-mortality frontier population of Baden-Württemberg in the south of western Germany. Baden-Württemberg was used alone because it is a large state (population 11 million) and had the highest *e*_5_ over the entire 1982–2015 period. Eastern and western populations were separated because of known differences in the epidemiological profiles of former East and West Germany,^12^ which we expected would have led to different trends in the age pattern of mortality decline.

More details on the data sources and methods used to calculate trends in regional *e*_5_ and national income across countries, as well as on the contour-decomposition methodology, can be found in the Online Supplementary Appendix A, available as Supplementary data at *IJE* online.

## Results

**Figure 1:**
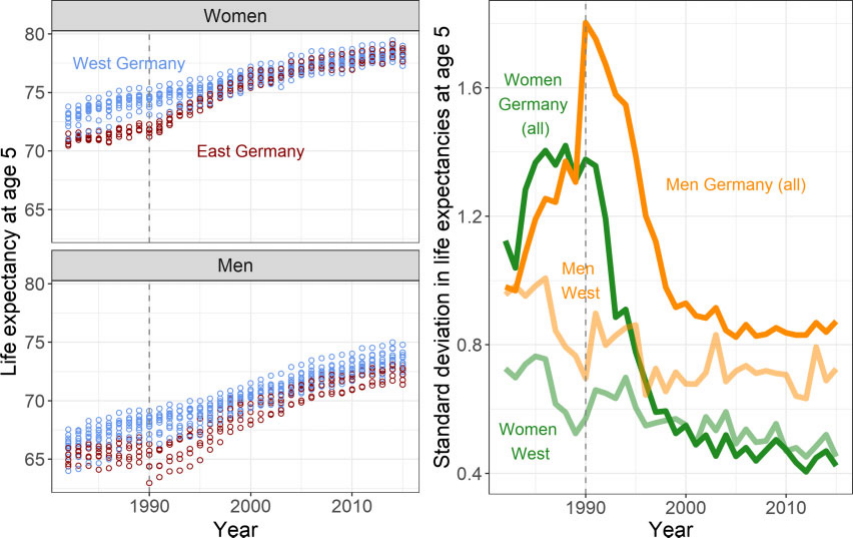
Life expectancy at age 5 years (*e*_5_) for each German state (left panels) and the standard deviation in *e*_5_ (right panel) by sex; 1982–2015 (in years). The lighter lines in the right panel refer to the subset of states in western Germany. The vertical line at 1990 demarks the German reunification—lines prior to 1990 are of a hypothetical united Germany.

Figure 1 shows that inequalities in *e*_5_ between German states changed substantially over the 1982–2015 period. The left panels overlay *e*_5_ values for each individual state from 1982 to 2015, with western and eastern German states marked separately. Among women, the *e*_5_ of eastern German states went from having *e*_5_ values below the lowest *e*_5_ in western German states prior to reunification, to having values that lie firmly within the middle of the state-level *e*_5_ distribution by around 2000. Among men, eastern German states had generally lower *e*_5_ levels throughout the period, with differences peaking shortly after reunification. The right panel of Figure 1 shows trends in the *SD* of *e*_5_ across states for western Germany and for Germany as a whole. Men and women started the period each with an *SD* in state-level *e*_5_ of about 1 year across Germany. From 1985 to reunification in 1990, *e*_5_ inequalities between states increased steeply. This was a period when *e*_5_ inequalities between western German states declined, whereas *e*_5_ differences between western and eastern German states increased. Immediately following reunification in 1990, eastern Germans experienced reductions in *e*_5_ (i.e. increases in mortality), especially pronounced among men. This caused state-level inequalities across the whole of Germany to reach their peak, before gradually falling back to their initial levels by around the mid-1990s for women and late 1990s for men. Women continued to experience declines in the *SD* of *e*_5_ in Germany, to a level of around 6 months in 2015, which is below that in western Germany and about half of its starting level in 1982. The *SD* in *e*_5_ for German men has been stable since the mid-2000s, at just under 1 year, and about 20% higher than the *SD* in western Germany.

**Figure 2:**
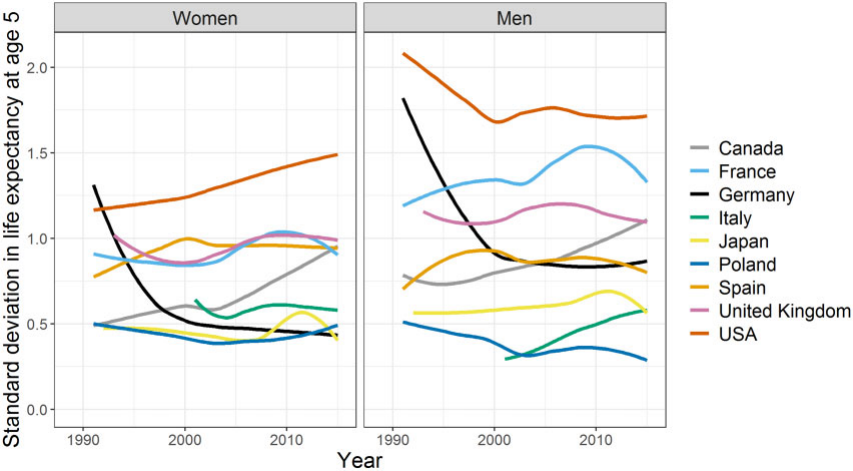
State-level inequalities in life expectancy at age 5 years in Germany and selected countries by sex, 1991–2015 (in years). (i) Data are smoothed using a Loess filter to make trends for each country more visible. Ruptures in the time series are caused by missing data points. (ii) Data sources, the method description as well as unsmoothed trends are available in the Online Supplementary Appendix A, available as Supplementary data at *IJE* online.

Figure 2 and Supplementary Appendix A Figure S1, available as Supplementary data at *IJE* online, contrast the German *SD* in state-level *e*_5_ to those in other large OECD countries from 1991 to 2016. In the early years following reunification, Germany had the highest regional mortality disparities for women and second-highest for men across all selected countries. However, within 10 years, these inequalities had decreased to average levels for men and to among the lowest observed levels for women, alongside those in Poland and Japan. Moreover, *SD* levels in Germany have either declined or been stable over the entire observation period. By contrast, the USA, Canada and Italy have all experienced sustained increases in *SD* since at least the turn of the century.

**Figure 3:**
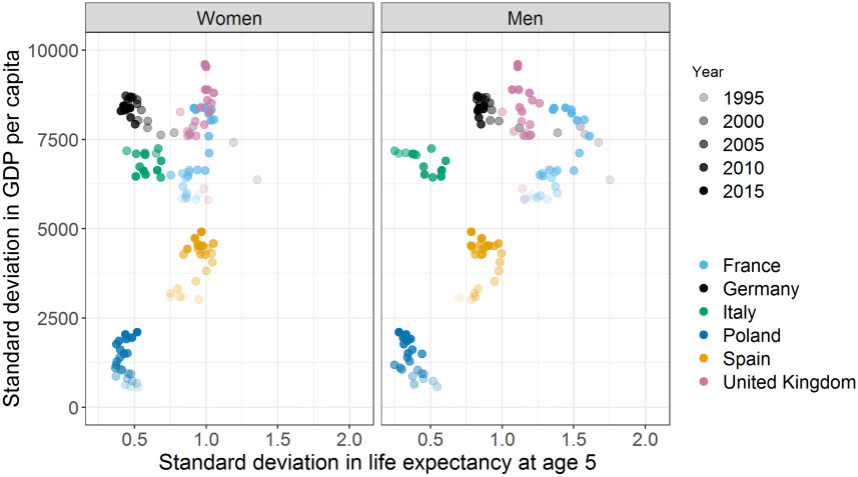
Association between state-level inequalities in life expectancy at age 5 years and state-level inequalities in GDP per capita in Germany and in selected European countries. (i) Each point refers to a country value in a given year between 1991 and 2015. (ii) Data sources and method description are available in the Online Supplementary Appendix, available as Supplementary data at *IJE* online). The standard deviation in GDP per capita is for both sexes combined.

Figure 3 shows that Germany consistently had among the highest *SD* in state-level per-capita GDP over the period from 1991 to 2015, despite its low *SD* in state-level *e*_5_, compared with other large European countries. In the 1990s, regional GDP differences were actually increasing, whereas mortality disparities were decreasing. Thus, the regional *SD* convergence in *e*_5_ in Germany occurred despite persisting regional economic disparities. We tested this relationship more rigorously in a series of panel regressions (see Supplementary Appendix B, available as Supplementary data at *IJE* online). We found that the change in per-capita GDP was associated with the change in life expectancy at the subnational level (Supplementary Appendix B Table S1, available as Supplementary data at *IJE* online); however there was no association found between the *SD* (GDP) and the *SD* (*e*_5_) across regions within countries (Supplementary Appendix B Table S2, available as Supplementary data at *IJE* online). To visualize the shifting regional variation patterns, Supplementary Appendix A Figure S2, available as Supplementary data at *IJE* online, displays Preston curves,^30^ i.e. the log-linear relationship between per-capita GDP and *e*_5_, across the German states for three time periods. Increasing state-level GDP is associated with higher life expectancy for men, but the slope of the association is nearly flat for women.

**Figure 4:**
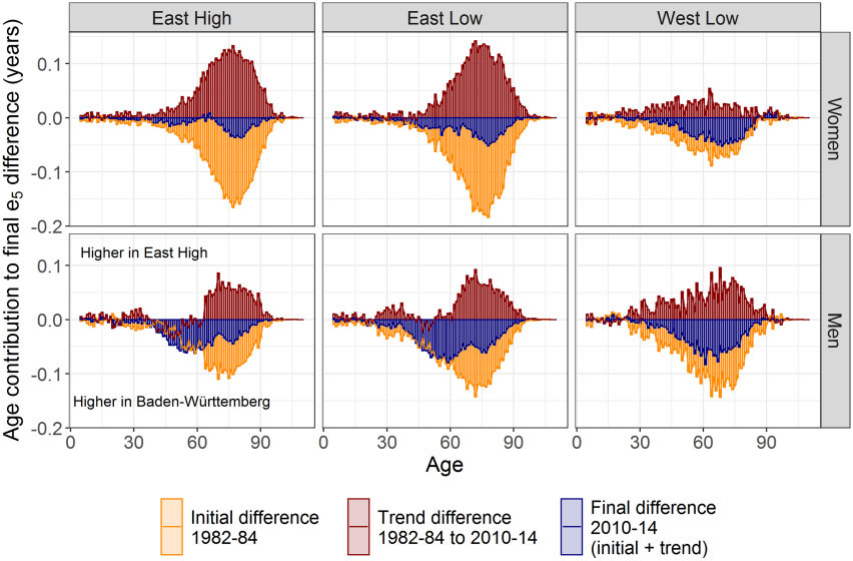
Contour decomposition of mortality differences over age, comparing population groupings with the frontier state *Baden-Württemberg* (BW). For details on population groupings (*East High*, *East Low*, *West Low*), see Online Supplementary Appendix A, available as Supplementary data at *IJE* online. The initial (1982–1984) and final (2010–2014) life expectancy at age 5 years values per state grouping were as follows: Women—*East High* (71.3, 78.4); *East Low* (70.9, 77.8); *West Low* (72.0, 77.7); *Baden-Württemberg* (74.1, 79.1). Men—*East High* (65.9, 72.6); *East Low* (64.9, 71.8); *West Low* (65.0, 72.6); *Baden-Württemberg (*67.8, 74.5).

Figure 4 presents contour-decomposition results, which identify the contribution of each age to the mortality convergence within Germany for the entire 1982–1984 to 2010–2014 period. In all panels, the *final* component (blue bars) is the contribution of different ages (in years of life) to the 2010–2014 gap in *e*_5_ between the state grouping and *Baden-Württemberg*. Positive *trend* components (red bars), e.g. among all populations above age 70 years, mean that mortality decline in these population groupings outpaced mortality decline in *Baden-Württemberg*, and led to converging *e*_5_. Trend components falling along the zero line, e.g. among women below age 40 years, in the comparison between *East Low* and *Baden-Württemberg* means that mortality decline was similar over these ages in *East Low* and *Baden-Württemberg*, and that these particular ages did not contribute to convergence or divergence in *e*_5_.

A few patterns are worth mentioning from these figures. First, younger ages contribute less to the *e*_5_ regional gaps among women than among men throughout the time period. In eastern states in particular, female gaps in mortality below age 65 years have almost been completely eliminated (blue bars), in part because they were small to begin with (orange bars). Second, men in *East Low* and *East High* experienced weaker or similar mortality declines to *Baden-Württemberg* below age 65 years over the period, but faster mortality decline above age 65 years (red bars). As a result, the contribution from ages below 65 years to the *e*_5_ gap between eastern states and *Baden-Württemberg* was mostly constant over time, whereas the contribution from ages above 65 years nearly halved (change from orange bars to blue bars). Third, for both men and women in *West Low*, the mortality reduction that contributed to converging *e*_5_ levels towards *Baden-Württemberg* was more uniform across age than it was in the eastern German states (i.e. red bars are more uniform in height). Finally, although the final gaps in *e*_5_ are similar for *East Low* and *West Low* women, and for *East High* and *West Low* men, the relative contributions of different ages to these gaps have not converged, particularly for men (i.e. the sum of the blue bars are similar, but age-specific profiles are not).

Splitting these results up by decade (Online Supplementary A Appendix Figure S3, available as Supplementary data at *IJE* online), the East–West differences are striking. In the East, mortality crisis in the late 1980s was followed by recovery in the 1990s and only moderate convergence to *Baden-Württemberg e*_5_ levels over the 2000s, whereas, in *West Low*, the narrowing of the *e_5_* gap with *Baden-Württemberg* has been more gradual. In the most recent decade (2000–2004 to 2010–2014), mortality decline was slower over mid-life in *East High* and *East Low* than in *Baden-Württemberg* among men, but not in *West Low*.

Overall, the main message from these contour decompositions is that women are converging in their age patterns of mortality regardless of East–West location, whereas different age patterns of mortality have emerged among men in the different groups of states, despite some convergence in life expectancy.

## Discussion

### Summary of findings

We demonstrated that, among large OECD countries, Germany went from having the highest internationally observed state-level inequalities in mortality upon reunification to today having the lowest inequalities for women and average levels for men. This convergence occurred despite persisting and comparatively large regional economic disparities. Age patterns of mortality are converging among women, but less so among men, between the longest- and shortest-living states, and across the former East–West political border.

### Interpretation

#### Regional inequalities in per-capita GDP and regional inequalities in life expectancy

Although we found that regional per-capita GDP levels were associated with regional life-expectancy levels within large European nations, which is consistent with existing findings on the relationship between mortality and economic conditions at the national^40^ and subnational regional levels,^11^^,^^31^ we did not find any association between inequalities in GDP and inequalities in life expectancy across subnational regions within these same countries.

In explaining these findings, first it is important to recall that the Preston-curve relationship^30^ was log-linear, meaning that increasing gains in GDP across populations corresponded to smaller gains in life expectancy. Given that the analysed countries have comparatively high levels of per-capita GDP, this might contribute to weaken the relationship between inequalities in GDP and inequalities in life expectancy. Our association plots also suggest that the distribution of income across subnational regions is unlikely to be one of the primary determinants of regional inequalities in life expectancy, at least in major European countries. This concords with findings at the individual level showing that the relationship between income inequality and inequalities in life expectancy is weaker in countries guided by a more extensive state-welfare system compared with the USA or the UK.^40^

Whereas regional contextual conditions do not appear to have a strong influence on mortality for the German state populations as a whole, it could be that this relationship differs by level of socio-economic status, which we were not able to test for. Findings from outside of Germany have shown the regional context to be particularly salient for individuals of low socio-economic status. Differences in mortality by education or income were larger for the most disadvantaged compared with least disadvantaged socio-economic groups across states,^41^ counties and commuting zones^42^ in the USA, and across European countries,^43^ suggesting that personal resources act as a buffer to living in countries or regions with less favourable health and social conditions, and weaker economic opportunities. Within US commuting zones, regional inequalities in mortality were strongly associated with factors that correlate to regional opportunities such as the fraction of immigrants, median house values and local government expenditures per capita, whereas factors associated with short-term economic conditions such as the unemployment rate or changes in the size of the labour force did not seem to be as important.^42^

However, if such contextual effects were important determinants of mortality at the German state level, we would have expected moderately high state-level inequalities in life expectancy, given the comparatively large regional differences in living conditions. This could result from compositional effects if poorer states contained a higher concentration of disadvantaged individuals, in part due to selective migration, as well as direct effects if per-capita income were causally associated with life expectancy or if disadvantaged individuals fared worse in poorer states.

#### The low regional inequality in life expectancy in Germany

Whereas this study was not designed to identify the mechanisms that generated comparatively low state-level inequalities in German mortality, we can speculate about a number of potential pathways that warrant future exploration. These include the impacts of fiscal transfers to ensure equality in social spending between the states, a diffused economy with many regional hubs and the patterning of health-adverse behaviour, particularly smoking.

Germany is a federation with much of the responsibility for health and welfare falling within the jurisdiction of the individual states. Nevertheless, Article 72, paragraph 2 of the German Constitution explicitly gives the national government the right to intervene and legislate on matters relating to the ‘establishment of equivalent living conditions throughout the federal territory’.^44^ In practice, this has meant substantial economic transfers from western Germany to the states of the former East Germany in the form of a 5.5% ‘solidarity’ surcharge levied on income tax, which continues to this day, almost 30 years after reunification. Also, a number of less wealthy western German states benefit from transfers from southern Germany, which might also have fostered convergence within western Germany.

Among European countries, economic activity is often strongly concentrated in capital regions. This is certainly the case for the UK and France, but not for Germany, which is characterized by a polycentric spatial economic structure with important economic centres dispersed across the country. Thus, whereas our results suggest that state-level variation in GDP was not an important factor driving state-level mortality inequalities in Europe, it could be that the skew in the GDP distribution across states plays a role.

The patterning of health behaviours, particularly smoking, has been shown to be a major driver of regional differences in mortality in the USA.^45^ Smoking is likely to be playing a lesser role in Germany. For one, smoking-attributable mortality is lower in Germany than in Anglo-Saxon countries.^46^ Second, women from the more deprived eastern states historically smoked less than those in the west, but differences were less pronounced between eastern and western men.^47^^,^^48^

It is important to point out that low state-level inequality in mortality is not the same as low inequality at the individual level. We showed that inequalities in GDP per capita between states in Germany have remained relatively stable since the 2000s, suggesting that most of the observed increasing income inequality in the country at the national level^49^ results from increasing within-state income inequality. If income inequality were an important determinant of population health in Germany, this would not be captured by analysis at the state level.

### Limitations

The shape and scale of population aggregation can influence the degree of between-district inequality.^50^ We have compared state-level inequalities at a high level of aggregation—what is known as NUTS 1 in Europe. In the German case, the states ranged in size from around 670 000 inhabitants in the city-state of Bremen to the nearly 18 million inhabitants of North Rhine-Westphalia. The latter has a larger population than that of all six eastern German states combined.

To test whether the comparatively low German regional inequality was also present at a smaller aggregation level (European NUTS 2, approximately 700 000 to 3 million inhabitants), we extracted *e*_5_ levels from the Eurostat database for the same subset of European countries (Online Supplementary Appendix A Figure S4, available as Supplementary data at *IJE* online). Germany had the lowest regional inequality among European countries for women, and the third lowest for men. The more favourable (egalitarian) ranking at the NUTS 2 compared with NUTS 1 level for German men might result from eastern regions being given smaller weight in a national regional-level comparison. At the NUTS 1 level, former East German regions contribute 6 out of 17 regions (35.2%) in an analysis including separated Berlin as we have here, whereas, at the NUTS 2 level, 8 out of 38 regions (21%) are in the former East Germany. Although Germany improved marginally in state-level-inequality rankings in moving from NUTS 1 to NUTS 2, in general, the country rankings were similar at both levels. Between the two levels of aggregation, we have less confidence in the NUTS 2 results because these data have not been corrected for the known numerator–denominator mismatch problems. Some of these problems are unique to the German context described earlier owing to the long intercensal period and a lack of harmonization. In addition, at the NUTS 2 level, frailty-selective migration is likely to gain relevance as a source of bias, when e.g. frail people leave metropolitan areas to move to and die in care facilities in surrounding regions.^51^

Thus, moving down the aggregation ladder, our results at NUTS 2 showed an *SD* in life expectancy that was about 10% higher, although still comparatively low internationally. A recent analysis at the NUTS 3 level determined a life-expectancy range between top and bottom quintiles of the 402 *Kreise* in Germany of around 5 years for men and a little over 2 years for women.^52^ Larger differences were found between counties in the USA^16^ and in England and Wales.^19^ The 5-year difference for men at NUTS 3 approximates the range in life expectancy that was found at the individual level between top and bottom income quintiles for both men and women in Finland.^38^ Comparable figures for Germany at the individual level are difficult to come by because of a lack of data that are reliable, sufficiently powered and capture the full age range.^53^ Censuses and large population surveys are not deterministically linked to death certificates. The little evidence that exists suggests that the German socio-economic gradient in mortality is growing and comparable to the gradient in other Western European countries.^53–57^

An additional concern could be whether the low *SD* in *e_5_* found for Poland and Germany might be because of their low life expectancies compared with France and Japan, and likewise that differences in the *SD* in per-capita GDP could be influenced by different levels between countries. As a robustness check, we replicated Figures 3 and 4 with the coefficient of variation, a relative measure of inequality (Online Supplementary Appendix A Figures S5 and S6, available as Supplementary data at *IJE* online). These results were similar for most of the observation period. Only the pattern for males became less exceptional when contrasted with GDP disparities at the end of the observation period.

## Conclusion

In summary, our results showing low variation in life expectancy across German states support Razum *et al.*’s^58^ argument that future health and social policies to further tackle mortality inequalities in Germany should be refocused away from small regional disparities in mortality towards the seemingly larger socioeconomic status (SES) disparities across individuals. To that end, results from the new German national cohort study^59^ may improve estimates of the socio-economic gradient in mortality. However, until the German census and population registers are linked to the death registries, the surveillance of mortality inequalities, particularly with regard to age and cause of death, will be unsatisfactory. It is nevertheless noteworthy that Germany reports low regional mortality disparities even though inequalities across SES groups appear to be comparable to other high-income countries.^56^

By gaining insight into the reasons for low and converging variation in life expectancy across states, the German example can demonstrate to other nations how massive sociopolitical change and long-term spatial socio-economic differences do not necessarily need to coincide with large spatial disparities in survival.

## Funding

This work was supported by the European Research Council [starting grant # 716323 to AvR], and funding from the Max Planck Society within the framework of the project ‘On the edge of societies: New vulnerable populations, emerging challenges for social policies and future demands for social innovation. The experience of the Baltic Sea States (2016–2021)’.

## Supplementary Material

dyz265_Supplementary_Data
