# Erratum to: Declining regional disparities in mortality in the context of persisting large inequalities in economic conditions: the case of Germany

**DOI:** 10.1093/ije/dyaa034

**Published:** 2020-03-10

**Authors:** Alyson A van Raalte, Sebastian Klüsener, Anna Oksuzyan, Pavel Grigoriev

First published online: 16 January 2020, *Int J Epidemiol* 2020;49:486–96. doi: https://doi.org/10.1093/ije/dyz265

The originally published version of this article contained errors in the numbering of all references from reference 40 onward. This has now been corrected and OUP apologizes for the error.

