# Supplementary material for: Declining regional disparities in mortality in the context of persisting large inequalities in economic conditions: the case of Germany
Source: Int J Epidemiol. 2020 Jan 16;49(2):486–96. doi: 10.1093/ije/dyz265 (PMC7266541; doi:10.1093/ije/dyz265)
Supplement: dyz265_Supplementary_Data [file dyz265_supplementary_data.pdf]

Online Supplementary Appendix A: Methods and Figures

1. Data and methods

1.1. German Regional HMD

Raw population counts for most states and years were extracted from the Genesis Online system (60). For the eastern German states (1982-1990) we obtained data from the state statistical offices who had reconstructed the population exposures backwards to the last East German census of 1981. These exposure data were adjusted as described in the main text. Birth counts were gathered from 1990 onward from the Genesis Online system of the German Federal Statistical Office. For the 1980s we obtained birth counts for western German states from statistical publications, and for eastern German states through data requests from the state statistical offices who had reconstructed the births backward.

Death counts originated from the statistical offices of the German states and the Federal Statistical Office of Germany. As not all states publish death statistics by single year of age up until the highest possible age, we used individual-level death register data from the Research Data Center of the Federal Statistical Office and the Statistical Offices of the German states (15) to move the open age category to ages 95 and above. In addition, we reconstructed death statistics for the eastern German states for the period 1980-1989 based on data derived from the official individual-level cause-of-death register of East Germany. The state of Berlin is separated into East Berlin and West Berlin to allow for continuous trends. A final hurdle was that it was no longer possible to distinguish East and West Berlin from official statistical data beyond 2001. We therefore used the estimation method implemented for the HMD (37) to separate births, deaths and population estimates into the two city parts.

Our calculations use the standard HMD methods protocol. Within the HMD project it is currently discussed to apply additional procedures for regional data in the HMD to more accurately measure mortality at higher ages. Thus, we cannot exclude that the life expectancy data which will be published in the German Regional HMD will slightly differ from the data which we used in this paper. After last methodological issues have been resolved and final data protection clearance has been obtained, the German Regional HMD will be made freely available online.

1.2. Remaining life expectancy at age 5 (*e5*) at the state level (international)

We used data from all large OECD countries with 30 million inhabitants or more. Excluded from the analysis were Korea, Mexico and Turkey because data were either unavailable or of a lower quality. We based our quality assessment on whether data at the national level were provided in the Human Mortality Database (HMD), which includes countries only when death registration is virtually complete (36). We used unaltered preexisting state-level life tables for all countries depicted in Figure 2 of the main text.

The United States of America

e5 was retrieved for each sex, province and year (1990 to 2011) from the United States Mortality Database (USMD) 1x1 (single age by single year) period life tables (61), which is based on HMD methodology.

Provinces of Canada

e5 was retrieved for each sex, province and year (1990 to 2011) from the Canadian Human Mortality Database 1x1 period life tables (62), which is based on HMD methodology. For years 2012 to 2015 we used life tables from Statistics Canada (63). Life expectancy values for the three territories (Nunavut, Yukon and the Northwest Territories) were not used because of small sample size.

Japanese prefectures

e5 was retrieved from the 5x5 period life tables of the Japanese Mortality Database for each of the 47 prefectures (64), which is based on HMD methodology. These tables were used because 1x1 life tables were not available. We retrieved life tables for years 1980-84, 1985-89, …., 2010-2014, and 2015. Data points for the standard deviation in e_5 are centred on the middle year; i.e. for 1980-84 is depicted as year 1982.

European countries

e5 by sex, year and state (or equivalent regional agglomeration, hereafter also described as state) were extracted for all EU countries with five or more states (Spain, France, Italy, Poland, and UK) from the abridged life tables available from Eurostat. This was done at the NUTS 1 level to make the agglomerations equivalent to the German states. French overseas territories were excluded.

As a robustness check, e_5 at the NUTS 2 level was also retrieved for all European countries including Germany. Mortality data for European countries were retrieved from Eurostat using the R-package eurostat (65).

Per capita GDP, and all contextual variables used for the panel data analysis were obtained from the European Union Urban Data Platform (66).

1.3. The Contour Decomposition method

When analysing the convergence in life expectancy between two populations, we are often faced with the question of how much the current difference is a legacy of the past, and how much is owing to different age-specific mortality trends. The recently developed contour decomposition method (39) allows us to answer precisely this question by splitting the age contributions of a conventional between-population decomposition into components reflecting (a) initial age-specific differences relating back to some point in time, and (b) a component relating to differences in age-specific mortality trends. In other words, this allows for a direct comparison of age-specific mortality trends given different initial mortality differences.

In a sense, the method works by adjusting the mortality contributions of the past e_5 difference to the new, typically lower mortality context. Specifically contour decomposition involves stepwise decomposing the age-specific mortality of two populations along an age-period contour. At each age, replacements of age-specific death rates were made in order from Baden-Württemberg in the final time period, back to Baden-Württemberg in the initial time period, then to the population of interest in the initial period and finally forward to the population of interest in the final period. After each replacement step, e_5 was recalculated. The end result was two vectors of age contributions to the current e_5 difference: an (averaged) trend contribution from the two populations and an initial between-population contribution. Importantly, these two vectors of age-specific contributions sum exactly to the age-specific contributions of the current e_5 difference obtained by conventional decomposition—a result that would not be possible by performing and combining three separate decompositions (the trend decompositions of changes in e_5 for each separate population plus one decomposition of initial differences in e_5).

To reduce the number of decompositions we grouped federal states into meaningful population groupings, based on their e_5 levels. The population groupings are: *East Low*—Mecklenburg-Western Pomerania (both sexes), Saxony-Anhalt (both sexes), and Brandenburg (males) / East Berlin

(females); *West Low*—West Berlin (both sexes), Saarland (both sexes), and Bremen (both sexes); *East High*—Saxony (both sexes), Thuringia (both sexes), and East Berlin (males) / Brandenburg (females).

An R-script to implement the contour decomposition method is freely available (67).

2. Figures

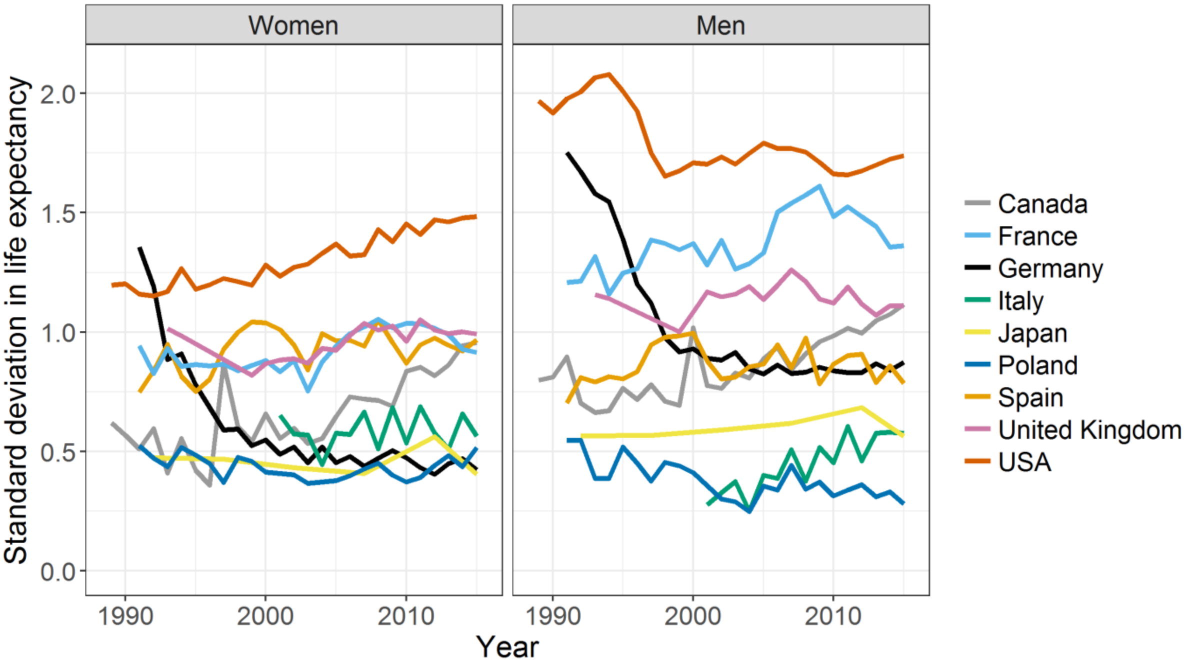

Figure S1: Unsmoothed state-level inequalities in life expectancy at age 5, 1990-2015 (in years)

Note: Ruptures in the time series are caused by missing data points.

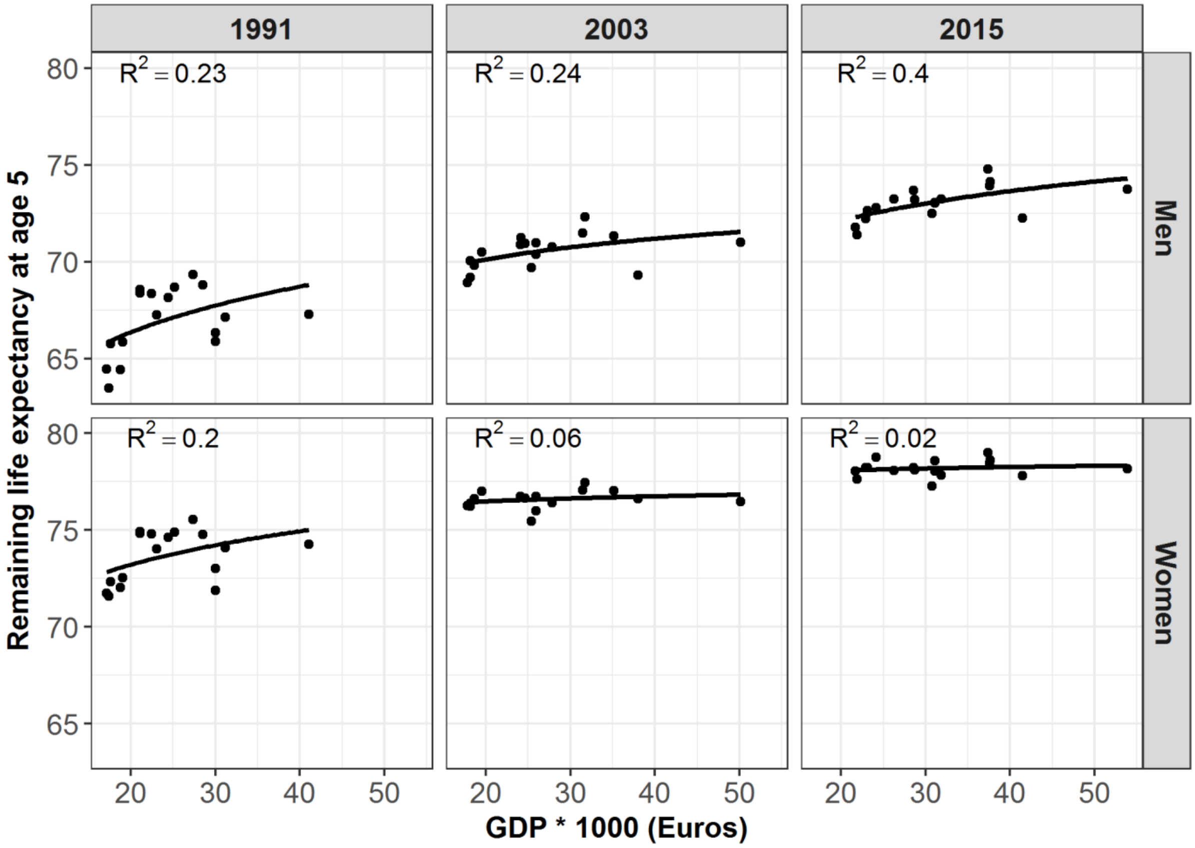

Figure S2: Preston curves showing the log-linear relationship between per capita GDP and remaining life expectancy at age 5 across German states at three different time periods

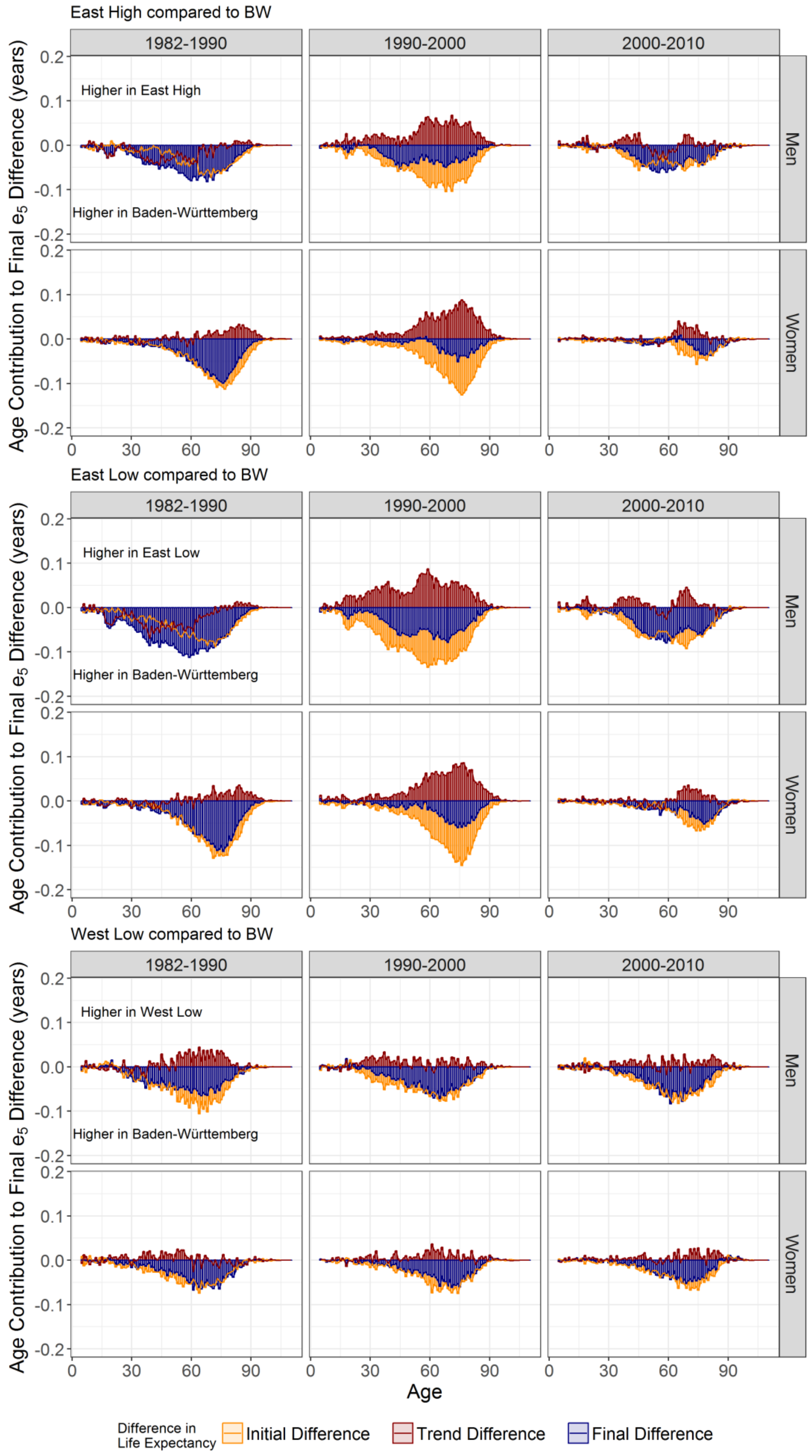

Figure S3: Contour decomposition of mortality differences over age, comparing population groupings with the frontier state Baden-Württemberg (BW) (by decade)

Notes: The population groupings are: *East High*—Saxony (both sexes), Thuringia (both sexes), and East Berlin (males) / Brandenburg (females); *East Low*—Mecklenburg-Western Pomerania (both sexes), Saxony-Anhalt (both sexes), and Brandenburg (males) / East Berlin (females); *West Low*—West Berlin (both sexes), Saarland (both sexes), and Bremen (both sexes). The initial (1982-1984) and final (2010-2014) e_5 values per state grouping were as follows: Women—East High (71.3, 78.4); East Low (70.9, 77.8); West Low (72.0, 77.7); Baden-Württemberg (74.1, 79.1). Men—East High (65.9, 72.6); East Low (64.9, 71.8); West Low (65.0, 72.6); Baden-Württemberg (67.8, 74.5).

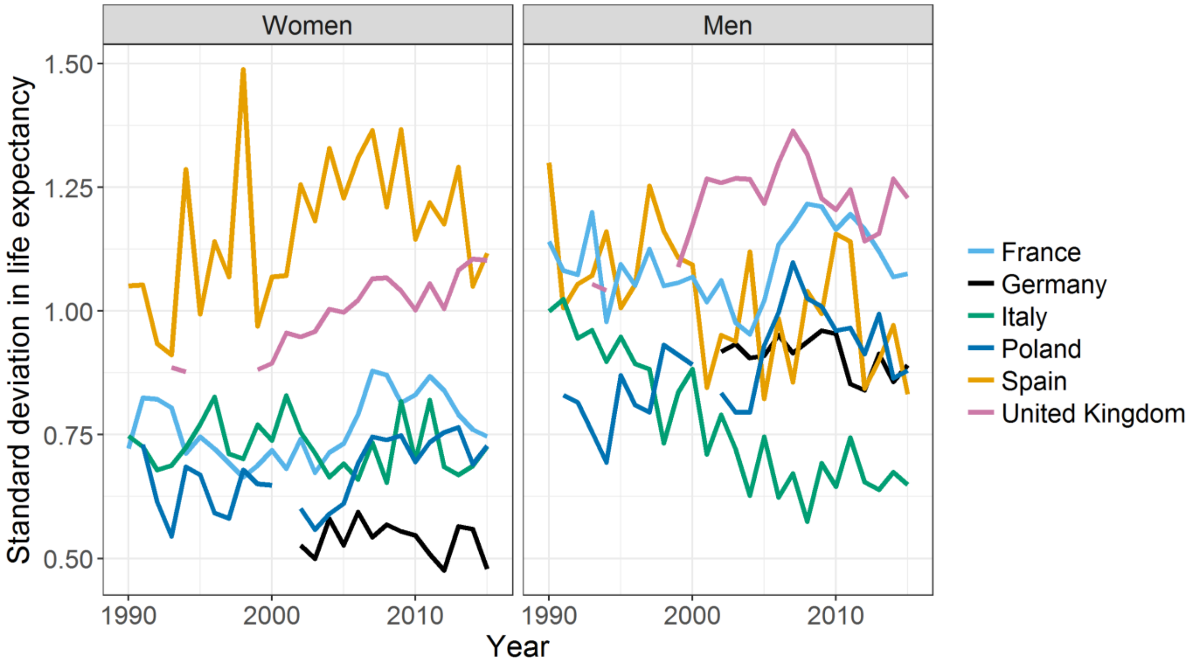

Figure S4: Robustness check of state-level inequalities in life expectancy at age 5 at NUTS 2 level for European countries, 1990-2015 (unsmoothed).

Note: Ruptures in the time series are caused by missing data points.

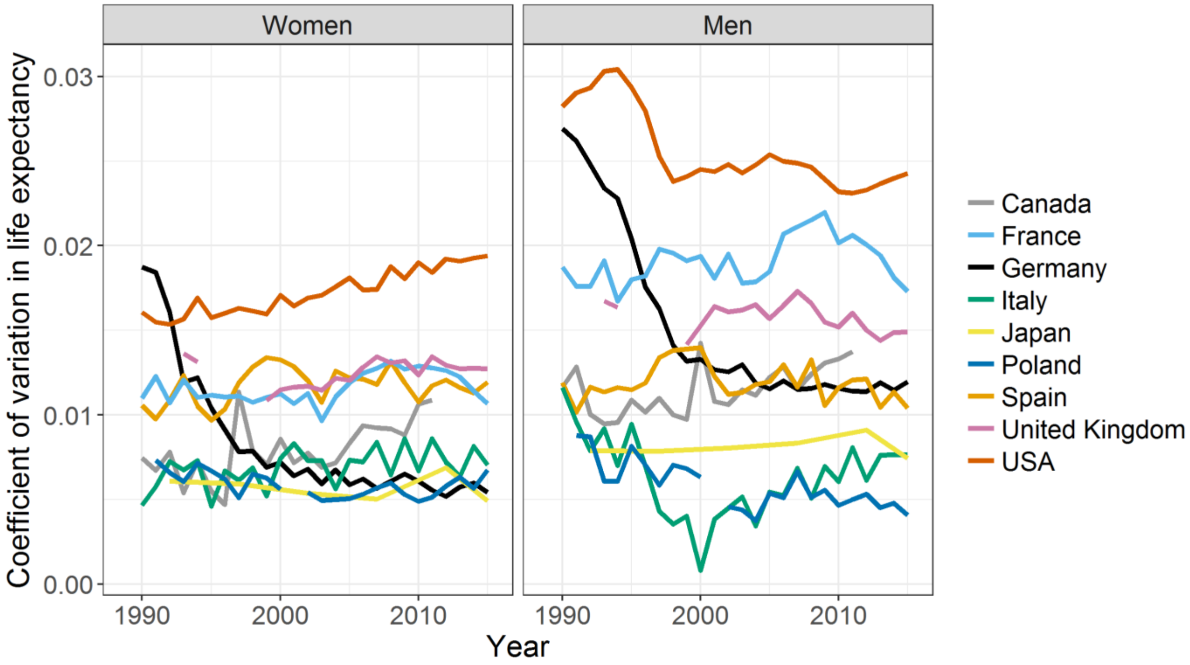

Figure S5: Coefficient of variation in life expectancy at age 5 levels in Germany and selected countries, 1990-2015.

Note: Ruptures in the time series are caused by missing data points.

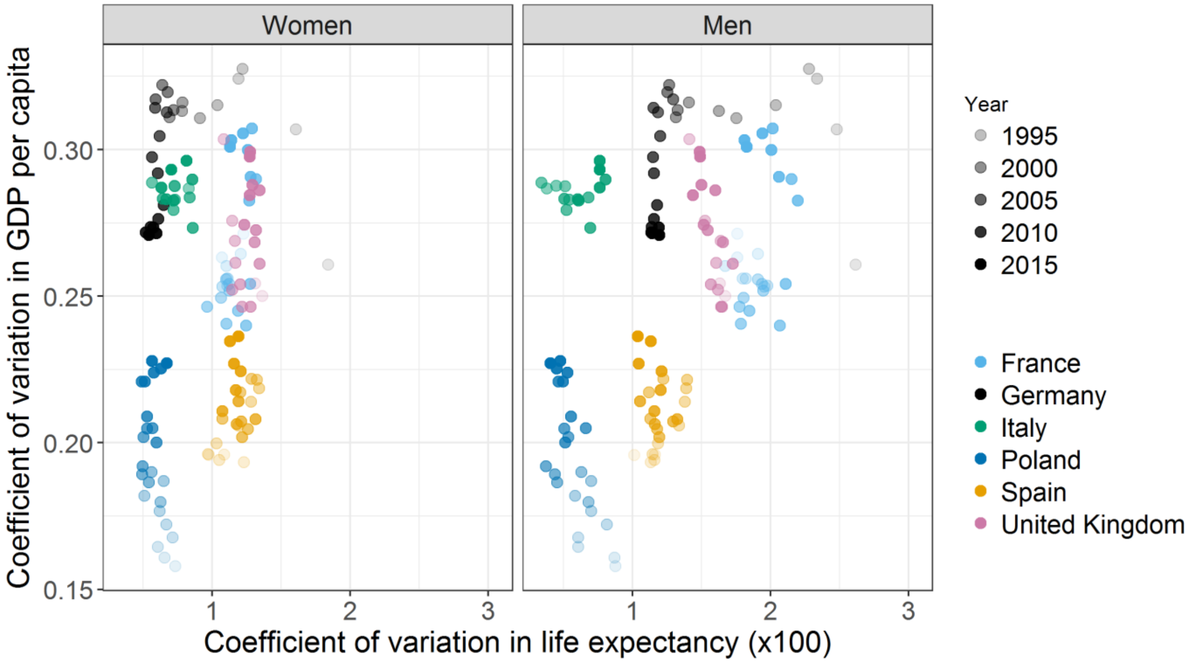

Figure S6: Associations between state-level inequalities in life expectancy at age 5 and GDP per capita in Germany and in selected European countries

Notes: 1) Each point refers to a country value in a given year between 1991 and 2016.

2) The coefficient of variation in GDP per capita is for both sexes combined.

Online Supplementary Appendix B

In order to examine statistically the association between GDP per capita and life expectancy at the regional level we used a panel regression model with fixed effects having the following general specification:

$$Y_{it} = X_{it} \cdot \beta_1 + \ldots + X_{kt} \cdot \beta_k + a_i + u_{it}$$

(1)

Where:

Y_{it} – dependent variable observed for entity *i=1,…, N* at time *t=1,…,T.*

$X_{it} \ldots X_{kt}$ – vector of time-variant independent variables;
k is the number of independent variables.

$\beta_{1\ldots}\beta_k$ – vector of the regression coefficients for independent variables.

a_i – the unobserved time-invariant effects (entity-specific intercepts).

u_{it} – the error term.

Our regression analyses consisted of two kinds of models: (Model 1) the relationship between GDP per capita and e_5, and (Model 2) the relationship between the standard deviation in GDP per capita and the standard deviation in e_5. Because of data availability constraints we were not able to make proper adjustments for confounders such as unemployment. The complete regional time series for this variable were only available for a few countries and shorter time periods.

Both models were run separately by sex using the regional data for the following six countries: France, Germany, Italy, Poland, Spain, and the UK. For each model we ran several diagnostic tests, and when applicable adjusted them for major disturbances such as heteroscedasticity and serial autocorrelation. We also tested a log transformation of the independent variable (GDP) given that the relationship between life expectancy and GDP has been found to be log-linear across countries at different levels of economic development (30). The use of a log transform did not influence the results. For the sake of a straightforward interpretation of the obtained regression

coefficients we preferred to use the absolute value of GDP per capita. All statistical analyses were performed in Stata 14.2 SE using the command *xtreg*.

In Model 1 we analyzed the association between per capita GDP and e_5 for European countries using region-specific fixed (time invariant) effects, with the data from the EU Urban Data Platform underlying Figure 3:

$$e5_{it} = GDP_{it} \cdot \beta_1 + a_i + u_{it} \qquad (2)$$

The basic model included GDP and e_5, and in a consistency check we replaced GDP with employee compensation as an alternative measure of economic development. The latter consists of wages and salaries and employers' social contributions, and thus might better approximate the income generated by private households. GDP, on the other hand, is the Total Gross Value Added, plus taxes less subsidies, and includes income generated both by companies and households.

The results obtained for Model 1 (Table S1) show that change in the absolute value of regional income (regional GDP per capita) is directly and consistently associated with change in regional life expectancy. For example, the regression coefficient obtained for German men implies that after adjusting major disturbances, an increase in GDP per capita by 1000 Euro is associated with an increase in e_5 by 0.65 [0.61;0.69] years. Models using employee compensation as an alternative measure of economic development were largely consistent with these results.

The notable exception here was Italy, where an inverse association between GDP and e_5 was found. In order to check to what extent the Italian data was influencing our results, we ran Model 1 (all countries) with and without Italy. The exclusion of Italy did not result in any notable changes.

Table S1

Results of panel regression with fixed effects obtained for Model 1

A. Males

	France	Germany	Italy	Poland	Spain	UK	All
GDP, 1000 Euro	0.72 [0.65;0.78]	0.65 [0.61;0.69]	-0.75 [-0.49;-0.61]	0.97 [0.91;1.02]	0.65 [0.56;0.74]	0.47 [0.42;0.52]	0.62 [0.59;0.65]
_cons	53.2 [51.6;54.9]	52.7[51.7;53.8]	92.3 [88.8;98.8]	58.8 [59.5;60.1]	59.8 [57.9;61.6]	58.4 [56.9;59.9]	56.2 [55.6;56.9]
R^2: within	0.7170	0.7248	0.6098	0.8986	0.5387	0.6252	0.6339
between	0.3480	0.3000	0.7743	0.6454	0.3385	0.1434	0.3790
overall	0.2946	0.2197	0.0245	0.5382	0.2832	0.1761	0.3549
F	483.97	1072.1	107.83	1214.35	195.05	357.03	2060.38
Prob>F	0.0000	0.0000	0.0000	0.0000	0.0000	0.0000	0.0000
corr (u_i,Xb)	–0.9093	–0.9333	–0.9838	-0.5100	–0.8143	–0.8999	–0.9018
rho	0.9413	0.9514	0.9847	0.8503	0.7162	0.9188	0.9464
Number of obs.	200	425	75	144	175	227	1246
Number of regions	8	17	5	6	7	12	55

Note: Figures in brackets denote 95% confidence interval.

B. Females

	France	Germany	Italy	Poland	Spain	UK	All
GDP, 1000 Euro	0.49 [0.44;0.54]	0.46 [0.43;0.49]	–0.49 [0.60;0.38]	0.89 [0.85;0.94]	0.48 [0.41;0.55]	0.33 [0.29;0.37]	0.45 [0.43;0.47]
_cons	66.4 [65.1;67.7]	64.0 [63.1;64.9]	91.3 [88.7;94.0]	68.8 [68.5;69.1]	69.8 [68.5;71.1]	66.9 [65.7;67.9]	66.5 [65.9;66.9]
R^2: within	0.6683	0.6662	0.5312	0.8934	0.5375	0.5987	0.5966
between	0.2610	0.1499	0.8564	0.3314	0.4348	0.2228	0.1218
overall	0.2373	0.1328	0.1706	0.5419	0.3726	0.2285	0.1505
F	384.81	812.45	78.19	1454.23	194.05	319.28	1759.72
Prob>F	0.0000	0.0000	0.0000	0.0000	0.0000	0.0000	0.0000
corr (u_i,Xb)	-0.9086	–0.9390	–0.9828	–0.4987	–0.7564	–0.8830	–0.8936
rho	0.9310	0.9422	0.9820	0.8744	0.6716	0.9028	0.9519
Number of obs.	200	425	75	144	175	227	1246
Number of regions	8	17	5	6	7	12	55

Note: Figures in brackets denote 95% confidence interval.

In Model 2 we tested the relationship between the standard deviation (SD) in GDP per capita and the SD in life expectancy at age 5 (e_5):

$$SD(e5)_{it} = SD(GDP)_{it} \cdot \beta_1 + a_i + u_{it} \tag{3}$$

The results (Table S2) suggest no association between SD in e_5 and SD in GDP per capita among our six observed countries. This was also the case in our robustness check where we replaced the SD in GDP with the SD in employee compensation.

Table S2

Results of panel regression with fixed effects obtained for Model 2

	Males	Females
SD (GDP), thousand Euro	0.03 [–0.22; 0.15]	-0.01 [–0.15; 0.14]
_cons	1.09 [0.03; 2.18]	0.79 [–0.05; 1.64]
R^2: within	0.023	0.002
between	0.392	0.175
Overall	0.354	0.116
F	0.21	0.02
Prob>F	0.666	0.885
corr (u_i,Xb)	-0.76	–0.48
rho	0.8977	0.7802
Number of obs.	133	133
Number of countries	6	6

Note: Figures in brackets denote 95% confidence interval.
